# The Natural Course of Non-Alcoholic Fatty Liver Disease

**DOI:** 10.3390/ijms17050774

**Published:** 2016-05-20

**Authors:** Luis Calzadilla Bertot, Leon Anton Adams

**Affiliations:** 1 School of Medicine and Pharmacology, the University of Western Australia, Nedlands, WA 6009, Australia; lcbertot@gmail.com; 2 Department of Hepatology, Sir Charles Gairdner Hospital, Nedlands, WA 6009, Australia

**Keywords:** nonalcoholic fatty liver, non-alcoholic steatohepatitis, fibrosis, hepatocellular carcinoma, cirrhosis, non-cirrhotic

## Abstract

Non-alcoholic fatty liver disease (NAFLD) is the most prevalent form of chronic liver disease in the world, paralleling the epidemic of obesity and Type 2 diabetes mellitus (T2DM). NAFLD exhibits a histological spectrum, ranging from “bland steatosis” to the more aggressive necro-inflammatory form, non-alcoholic steatohepatitis (NASH) which may accumulate fibrosis to result in cirrhosis. Emerging data suggests fibrosis, rather than NASH *per se*, to be the most important histological predictor of liver and non-liver related death. Nevertheless, only a small proportion of individuals develop cirrhosis, however the large proportion of the population affected by NAFLD has led to predictions that NAFLD will become a leading cause of end stage liver disease, hepatocellular carcinoma (HCC), and indication for liver transplantation. HCC may arise in non-cirrhotic liver in the setting of NAFLD and is associated with the presence of the metabolic syndrome (MetS) and male gender. The MetS and its components also play a key role in the histological progression of NAFLD, however other genetic and environmental factors may also influence the natural history. The importance of NAFLD in terms of overall survival extends beyond the liver where cardiovascular disease and malignancy represents additional important causes of death.

## 1. Introduction

The prevalence of non-alcoholic fatty liver disease (NAFLD) parallels that of obesity, which has steadily risen throughout the world over the past thirty years [1]. The natural history of NAFLD in some individuals, is to progress to end-stage liver disease. Thus, NAFLD is projected to become the leading cause of liver related morbidity and mortality within 20 years and a leading indication for liver transplantation in the next few years [2]. Although the potential for NAFLD to progress to both cirrhosis and hepatocellular carcinoma (HCC) has been recognized for decades, more recent insights have helped define the magnitude of risk of progression and led to the understanding that NAFLD is a leading cause of cryptogenic cirrhosis [3,4,5,6]. More recently, accumulating evidence has also led to the hypotheses that even steatosis and mild inflammation can progress to fibrosis and HCC [7,8,9]. Nevertheless, the natural history of NAFLD remains incompletely defined, with key knowledge gaps including the lack of understanding behind the substantial inter-individual variation in disease progression and outcomes and understanding of the links between NAFLD and HCC. In this review we provide an up to date assessment of the natural history of NAFLD and emerging evidence that may impact the management of this disease in the future.

## 2. Histological Course of Non-Alcoholic Fatty Liver Disease (NAFLD)

Non-alcoholic fatty liver disease (NAFLD) encompasses a histological spectrum from non-alcoholic fatty liver (NAFL), which is characterized by steatosis with no or minor inflammation, to non-alcoholic steatohepatitis (NASH) where inflammation and ballooning is present, with or without fibrosis. The natural history of NASH tends to parallels the more aggressive histological picture, with prospective cohort studies demonstrating a higher rate of morbidity and mortality compared to NAFL, particularly when fibrosis is present [10,11]. Nevertheless, a limited amount of high-quality prospective data on the progression of NAFLD exists, particularly in the primary-care setting, where routine biochemical indices do not accurately reflect disease activity or progression. Paired liver biopsy studies from tertiary care cohorts provide valuable information however are limited in their generalizability due to selection bias.

**Figure 1:**
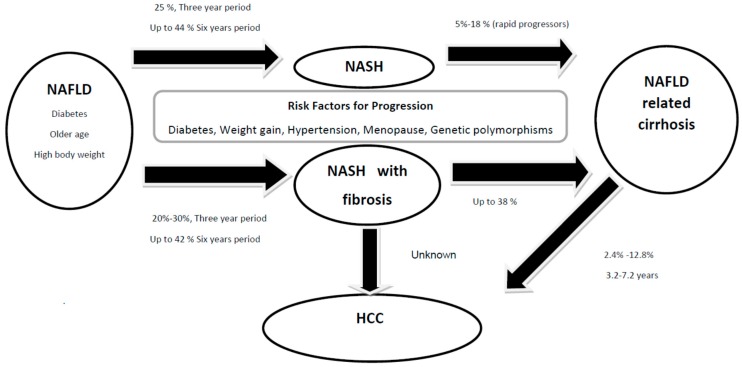
Progression of non-alcoholic fatty liver disease (NAFLD) to non-alcoholic steatohepatitis (NASH) with or without fibrosis, cirrhosis, and hepatocellular carcinoma. Data adapted form [7,8,9] and [24].

At least 12 studies have analysed the progression of steatosis, steatohepatitis, and fibrosis in NAFLD cohorts by utilizing paired liver biopsies [7,9,11,12,13,14,15,16,17,18,19,20]. These studies suggest that one third of patients with NAFL and NASH have progressive fibrosis and 20% will have some regression over an average follow-up between 2.2 and 13.8 years [7,9,11,12,13,14,15,16,17,18,19,20,21,22,23]. The rate of fibrosis progression is characteristically slow with a recent meta-analysis determining an average progression of one stage to take 7.7 years [24]. Nevertheless, the rate of progression is twice as high in NASH subjects and a sub-group of both NASH and NAFL patients may progress rapidly from no fibrosis to advanced fibrosis over an average six year period [8,9]. In contrast to fibrosis progression over time, features of steatosis, inflammation and ballooning tend to reduce which is paralleled by reduction in amino-transaminase levels [12]. Factors that may influence the histological progression of NASH are illustrated in Table 1, Figure 1 and outlined below.

## 3. Predictors of Progressive Fibrosis in Non-Alcoholic Steatohepatitis (NASH)

### 3.1. Sex

No consistent relationship between sex and fibrosis has been found in NASH, with cross-sectional studies reporting conflicting findings [25,26]. The relationship between sex and fibrosis may be influenced by menopausal status; cross-sectional studies have found men and post-menopausal women to have a higher risk of fibrosis compared with pre-menopausal women, and early menopause and duration of menopause to be associated with a higher risk of fibrosis [27,28].

### 3.2. Race and Ethnicity

Hispanic patients have an increased prevalence of NAFLD compared to Caucasians; however, there appears to be no difference in degree of liver injury between these ethnic groups [29,30]. In contrast, Asian patients may be prone to more severe histological changes including ballooning, whereas African-Americans may have less severe histology, although factors such as diet may be confounding this relationship [31,32,33].

### 3.3. Genetic Polymorphisms

Polymorphisms in the *PNPLA3* and *TM6SF2* genes are common in the general population with minor allele frequencies of 20%–50% and 10%, respectively [34]. The rs738409 and rs58542926 single nucleotide polymorphisms (SNP’s) of these respective genes have been identified by genome-wide association studies to be associated with an increased risk of NAFLD, as well the presence of more severe liver histology (*i.e.*, NASH and fibrosis) [34,35,36,37,38]. One study of over 1000 individuals with biopsy proven NAFLD, demonstrated these SNPs were associated with a 40% to 88% increased risk for advanced (F2-4) fibrosis after adjustment for age, sex, and metabolic variables [34]. Similarly, a SNP in the *IFNL4* gene, which is associated with response to interferon based treatment in chronic hepatitis C, has also been associated with fibrosis in NAFLD and has been amalgamated into a predictive score in conjunction with other clinical factors [39].

### 3.4. Age

Cross-sectional studies have demonstrated increasing age to be consistently associated with more severe fibrosis in NASH patients; however, this may reflect the cumulative sum of metabolic exposures and longer duration of NAFL/NASH in these populations [26,40,41]. In contrast, longitudinal studies have not consistently demonstrated age to impact the rate of fibrosis progression [24].

### 3.5. Metabolic Features

Diabetes and obesity have demonstrated to be predictive of a higher rate of fibrosis progression in some but not all longitudinal studies [7,8,9,12,22]. An increase or decrease in body mass index over time, has been associated with progression or resolution of liver fibrosis respectively in NAFLD patients and the emergence of diabetes also appears to parallel fibrosis progression, whereas improved glycemic control parallels fibrosis improvement [7,8,9,22]. One meta-analysis examining the full spectrum of NAFLD found hypertension to be a risk factor for fibrosis progression, however an earlier meta-analysis limited to NASH patients did not [24,42].

### 3.6. Histological Factors

The degree of hepatic steatosis does not appear to predict disease progression in NASH. The degree of inflammation however, has been associated with progression to advanced fibrosis in a meta-analysis, but not in any single cohort study [24].

## 4. Clinical Course of NAFLD

### 4.1. Liver Cirrhosis, Decompensation, and Liver Related Mortality

Overall, the risk of progression to cirrhosis and decompensation in NAFLD patients is low with a population based study demonstrating an incidence of 3.1% for both end-points over a mean 7.6 year follow-up [43]. Nevertheless, the risk of cirrhosis may be underestimated given the lack of systematic evaluation for its development in the community.

The risk of progression to end-stage liver disease is influenced by the severity of underlying liver histology; the majority of patients with NAFLD have simple steatosis, however, up to 30% of patients may have NASH [44] and are at greater risk. Several studies with up to 20 years follow-up, have demonstrated that the risk of progression to cirrhosis in patients with simple steatosis is between 0% and 4% [6,45,46]. In contrast, estimates of progression to cirrhosis in NASH patients varies with 10% developing decompensated liver disease over 13 years [11] and 25% developing cirrhosis over nine years [11]. The rate of progression is clearly heavily influenced by the underlying fibrosis stage, with NASH patients without fibrosis at significantly lower risk compared to those with advanced fibrosis. Progression to advanced fibrosis and cirrhosis is not uniform in all patients and metabolic factors such as presence of glucose intolerance and Type 2 diabetes mellitus (T2DM) may play a key role in this progression [47,48].

Once cirrhosis has developed, the risk of developing a major complication of portal hypertension is 17%, 23%, and 52% at one, three, and 10 years, respectively [49]. The survival of patients with NASH cirrhosis falls markedly once decompensation occurs, with a median survival of approximately two years [50]. Today, NAFLD is the second commonest etiology for listing for liver transplantation, and on the trajectory of becoming the most common cause [51,52,53,54]. Notably, the burden of NAFLD related cirrhosis may be under-estimated, as the histological signs of steatohepatitis may no longer be present at the cirrhotic stage of disease [55]. Caldwell *et al.* noted that a large proportion of patients with cryptogenic cirrhosis had been exposed to metabolic risk factors [4] and almost half of the cases of “cryptogenic” cirrhosis could ultimately be traced to the end-stage evolution of NASH [39].

Compared with individuals of the general population of the same age and gender, those with NAFLD have a lower than expected survival, at a standardized mortality ratio from 1.34 to 1.69 according to American and Swedish studies [11,12]. The increase in mortality hazard is likely in part, to be related to increased liver related mortality, with liver death the third commonest cause of death in two large cohort studies [11,12].

### 4.2. Non-Liver Related Death

NAFLD is associated with a significantly higher overall mortality compared to the age and sex-matched general population, which in part is likely related to excess vascular as well as liver-related death. Cross-sectional population-based studies and meta-analysis have demonstrated NAFLD to be independently associated with predictors of cardiovascular disease including endothelial dysfunction, arterial stiffness and myocardial dysfunction [56,57,58,59]. Notably, NAFLD results in hepatic insulin resistance, increased fasting glucose levels and an atherogenic lipid profile [60], and NASH is associated with increased levels of inflammatory pro-atherogenic cytokines, hyper-coagulable factors, and adhesion molecules [61].

Supporting these observations, analysis of over 11,000 participants in the NAHNES study conducted between 1989 and 2004 with median follow up of 14.5 years, demonstrated increased (69%) overall mortality in NAFLD patients with advanced fibrosis assessed by means of NAFLD fibrosis score, APRI and FIB 4. The increase in mortality in this subgroup was largely driven by cardiovascular disease (CVD) (adjusted hazard ratio 2.7 to 3.5) [62]. Other cohort studies have suggested that other sub-groups of NAFLD patients, such as those with type 2 diabetes [63] or men with an elevated gamma-glutamyl transpeptidase [57], may have an increased risk of CVD events compared to subjects without NAFLD. Thus, there may be other genetic or environmental factors that modify the association between NAFLD and CVD. Lastly, severity of liver histology may stratify risk of cardiovascular mortality with Ekstedt and colleagues demonstrating that subjects with simple steatosis did not have an increased risk of all-cause death or death related to CVD, but those with NASH were twice as likely to die from CVD compared to the reference general population (15.5% *vs.* 7.5%) over a mean follow-up period of 13.7 years [64].

## 5. Evolving Concepts

### 5.1. NAFL vs. NASH

A pioneer research published in 2006 compared the levels of serum concentrations of transforming growth factor-beta1 (TGF-β1) a marker of fibrosis, and ferritin between NAFL, and NASH patients [40]. No differences in the serum levels of TGF-β1 and ferritin were found between NAFL and NASH groups. Authors suggested that both NAFLD spectrums share common aspects regarding their progression and NAFL perhaps not so benign. Recent reports suggest NAFL may not be as benign as previously thought, with evidence of progression to advanced fibrosis, challenging the paradigm that risk of fibrosis progression is dichotomized according to the presence or absence of NASH (Table 2). Wong *et al.* [7] reported in a prospective study of paired liver biopsies taken a median three years apart, that 58% of patients with histological NAFLD activity score (NAS) <3 (*i.e.*, non-NASH) increased their activity score and 28% had fibrosis progression at three years. Fibrosis progression was seen in 20% to 30% of patients with both low and high NAS scores. Twenty-three per cent of patients with simple steatosis developed NASH in 3 years.

A retrospective study analysing a database of 70 NAFLD patients with paired biopsies showed that patients with NAFL can evolve towards well-defined steatohepatitis, and in some of them, bridging fibrosis after a follow-up of less than 5 years. The presence of mild lobular inflammation or any amount of fibrosis substantially increased the risk of histological progression in the mid-term while those with steatosis alone are at lowest risk [8]. More recently McPherson *et al.* [9] in the DELTA study included 108 patients with paired liver biopsies over a median of 6.6 years; they found overall that NAFLD had a variable natural history with 42% of patients having progression of fibrosis and 18% having regression of fibrosis. Of those with NAFL at the index liver biopsy, 44% progressed to NASH and 37% had progression of fibrosis, including 6 patients who developed stage three fibrosis.

Lastly, Singh *et al.* conducted a systematic review and meta-analysis of 11 studies involving 411 patients with paired liver biopsies [24]. Patients with both NAFL and NASH were found to develop progressive liver fibrosis, although the rate of fibrosis progression was higher in those with NASH than NAFL (one-stage progression over 7.1 years *vs.* 14.3 years, respectively). Collectively, these studies suggest that overall NAFL has a more indolent rate of progression than NASH; however, there is considerable heterogeneity, with one quarter of NAFL patients developing bridging fibrosis over a relatively short time period. Currently, reliable histological and clinical predictors of disease progression are lacking, however it appears that worsening metabolic disease (weight gain, diabetes) frequently parallels the histological progression [8,9].

### 5.2. Prognostic Significance of NASH vs. Fibrosis

The prognosis of an individual patient with NAFLD is highly variable. A greater likelihood of progressive disease was initially described in those patients with NASH, which is often defined according to the NAFLD activity score (NAS score). The NAS is the unbalanced sum of steatosis, ballooning, and lobular inflammation [10], and was originally developed as a tool for assessing efficacy in clinical trials, however has been applied more widely to define NASH and assess histological activity.

Recent evidence coming from prospective cohort studies suggest that fibrosis predicts liver and non-liver related mortality more reliably than NAS or its individual components [64,65,66]. A study by Younossi *et al.* of 209 NAFLD patients with a median follow up of 12 years found that advanced fibrosis was the only histological lesion independently associated with liver-related mortality (hazard ratio = 5.68, 95% confidence interval (1.5–21.4) [66] More recently Ekstedt and colleagues analysed a cohort of 229 biopsy proven NAFLD patients followed for a mean of 26.4 years [62]. Overall, NAFLD patients had an increased mortality compared with a matched reference population with NAFLD subjects with fibrosis stage three or four at baseline having the worst prognosis (HR 3.3, CI 2.27–4.76, *p* < 0.001). In contrast patients with a high NAS (5–8) without severe fibrosis did not have increase mortality compared with reference population. Finally, Angulo *et al.* conducted an international multicentre cohort study to determine the long term prognostic significance of histologic features of NAFLD [65]. This study confirmed that fibrosis stage rather than NASH, was the most important histological feature associated with overall survival and liver-related complications. Notably, even patients with mild fibrosis (stage 1) had a greater hazard for overall mortality compared to those with no fibrosis, although only those with moderate fibrosis (stage F2 and above) had a greater risk of liver related complications such as ascites, encephalopathy or varices. These studies emphasize the need to assess fibrosis routinely in all patients with NAFLD to assess their prognosis and, thus, need for monitoring and liver targeted treatment.

## 6. Hepatocellular Carcinoma (HCC)

### 6.1. HCC in NAFLD

HCC is the six most common cancer worldwide, the third most common cause of cancer related death and has a globally rising incidence [67,68]. Several studies have demonstrated an association between MetS, T2DM as well as obesity, with HCC, suggesting that NAFLD is playing a significant role in the rising incidence of HCC [67,69,70]. The potential mechanisms relating MetS, obesity, diabetes, NAFLD, and HCC, particularly in the absence of cirrhosis, are probably related to the pathogenesis of the underlying disease rather than to fibrosis alone. A fertile soil for liver carcinogenesis include insulin resistance and hepatic steatosis promoting adipose tissue-derived inflammation, hormonal changes (adipokines), oxidative stress, lipopoxicity, and stimulation of insulin-like growth factor [21,69,70]. Gut microbiome, diet, and genetics are increasingly important factors. Intestinal dysbiosis associated with obesity modify the gut microbiome and promotes the release of endotoxins [22]. High-fat diets and high fructose intake can worsen the cytokine pattern and promote lipoperoxidation [70]. Genetics contributes to increase the risk of HCC, mainly through the *PNPLA3* rs738409 variant [23].

NASH was found to be the third most common risk factor for HCC in a U.S. veterans population of 1500 with HCC diagnosed over a six year period [71]. Nevertheless, HCC remains an uncommon complication of NAFLD and heavily influenced by the presence or absence of underlying cirrhosis. For example, one Japanese study of 6508 individuals with ultrasound diagnosed NAFLD, found the HCC incidence to be only 0.2% after eight years, however subjects with advanced fibrosis determined by the AST-Platelet Ratio Index, had a 25-fold increase in risk [72]. Of concern however, are emerging reports of the development of HCC in non-cirrhotic patients; however, the magnitude of this risk remains to be defined [73,74,75].

### 6.2. HCC in NAFLD Cirrhosis

The cumulative incidence of HCC in NASH cirrhosis ranges between 2.4% and 12.8% over a 3.2–7.2 year period, and the cumulative HCC mortality in NAFLD/NASH cohorts is 0%–3% over 5.6–21 years [76]. A large series of 195 NAFLD cirrhosis patients from the Cleveland Clinic found the annual incidence of HCC to be marginally lower than a comparative population of hepatitis C cirrhosis patients (2.6% *vs.* 4.0%, *p* = 0.09) [77]. These findings have been replicated in other American and Japanese cohorts [50,78]. All these studies performs a defined protocol excluding other etiologies of HCC including Hepatitis C and Hepatitis B virus infection. Risk factors for HCC development in the NASH population included diabetes, age, any previous alcohol consumption and the presence of intra-hepatic iron [77,79]. Interestingly, the use of metformin in patients with type 2 diabetes has been associated with a reduced risk of HCC, suggesting that this risk factor may be modifiable [80].

Once HCC develops in NAFLD cirrhotic patients, survival appears to be shorter survival than patients with HCV-HCC [81]. This may be related to patients with HCC resulting from NAFLD being older, having larger tumours, and being less likely to be diagnosed by surveillance compared with HCC caused by viral hepatitis [82,83,84]. Nevertheless, among patients that have liver function and tumours eligible for curative HCC treatment, overall survival is similar or better that comparable patients with hepatitis C or alcohol induced cirrhosis [81,84].

### 6.3. HCC in NAFLD without Cirrhosis

The development of HCC in non-cirrhotic patients with NAFLD is increasingly reported with cross-sectional studies demonstrating between 15% and 50% of cases being diagnosed without cirrhosis [73,81,85,86]. Moreover, HCCs have been reported to arise in subjects without evidence of NASH or fibrosis but just simple steatosis [83]. A minority of these cases may be related to transformation of hepatic adenomas, whereas the majority appear to be related to risk factors for NAFLD, namely the MetS, obesity, and diabetes [87]. Several studies have also suggested that HCC originating in non-cirrhotic patients with NASH and/or the metabolic syndrome, are more likely to be male [85,86,87].

Not surprisingly, HCC associated with non-cirrhotic NAFLD is less likely to be detected during surveillance and thus is more likely to be more advanced when compared to HCC in cirrhosis patients [68,81,84]. Nevertheless, survival is equivalent or better in non-cirrhotic NAFLD patients when compared to subjects with cirrhotic-HCC, likely due to preserved liver function.

## 7. Conclusions

NAFLD is common in the general population, however the natural history and impact on patient morbidity and mortality is widely divergent. Metabolic factors, such as diabetes, obesity, and hypertension, as well as common genetic polymorphisms in the *PNPLA3* and *TM6SF2* genes, influence the severity of underlying liver histology and, thus, are likely to impact on risk of developing cirrhosis and HCC. Recent studies have demonstrated NAFL in addition to NASH, may lead to progressive fibrosis and have emphasized the importance of fibrosis level in determining future mortality risk. A greater understanding of the factors that alter the natural history of NAFLD will lead to better prognostication and targeting of NAFLD populations at greatest risk for specific therapies.

## Figures and Tables

**Table 1:** Risk factors for fibrosis progression in non-alcoholic fatty liver disease (NAFLD): Results from paired liver biopsy studies.

Study Author, Year	*n*	Mean/Median (Standard Deviation or Range) Follow up in Years	Predictors of Fibrosis Progression	Odds Ratio (95% CI)
Adams (2005)	103	3.2 (±3.0)	Diabetes	1.48
			Fibrosis stage	0.80
			BMI (per kg/m^2^)	1.04
Fassio (2004)	22	4.3 (3.0–14.3)	Obesity	NR
Argo (2009) *	221	5.3 (1.0–21.3)	Age	0.98 (0.96–0.99)
			Any inflammation at initial biopsy	2.5 (1.4–4.3)
Wong (2010)	52	3, NR	High LDL	2.7 (1.2 to 6.1)
			High waist circumference	1.3 (1.1 to 1.5)
Sorrentino (2010)	149	6.4	Fibronectin immunohistochemistry	14.1 (6.9–32.3)
			Hypertension	4.8 (2.7–18.2)
			HOMA-IR > 10	1.9 (1.6–12.1)
Pais (2013)	70	3.7 (±2.1)	^ steatosis grade	NR
Chan (2014)	35	6.4 (±0.8)	nil	-
McPherson (2014)	108	6.6 (1.3–22.6)	At baseline biopsy FIB 4 score	2.1 (1.1–3.9)
			At follow up biopsy FIB 4 score	3.1 (1.4–6.8)
			Diabetes	6.25 (1.88–20)
Singh (2015) **	411	NR	Hypertension	1.94 (1.00–3.74)
			Low AST:ALT ratio at baseline biopsy	−0.08 (−0.16–0.00)

* A systematic review comprising 10 studies; ** A meta-analysis including 11 cohort studies; ^ Progression defined by progression from non-alcoholic fatty liver (NAFL) to non-alcoholic steatohepatitis (NASH), occurrence of bridging fibrosis or at least one point increase in the NAFLD activity score (NAS) score from <5 to 5, or greater; NR = Not reported; HOMA–IR = homeostasis model of assessment-insulin resistance.

**Table 2:** Fibrosis stage as predictor of liver related complications, death, and overall mortality.

Study Author, Year	NAFLD Patients (*n*)	Mean Follow up (Years)	Histological Subgroup (*N*)	Cirrhosis and Liver Related Complications HR	Liver Related Mortality HR	Overall Mortality HR
Ekstedt *et al.*, 2015	229	26.4	NAS 0–8	10.8	3.3	3.28
			Fibrosis stage 3–4			
			*n* = 16			
Younossi *et al.*, 2011	257	12.1	Fibrosis stage 3–4	-	5.68	-
			*n* = NR			
Angulo *et al.*, 2015	619	12.6	Fibrosis stage			
			F1 *n* = 141	* 2.38	-	1.88
			F2 *n* = 85	* 7.51	11.2	2.89
			F3 *n* = 53	* 13.78	85.79	3.76
			F4 *n* = 18	* 47.46	-	10.9

* Results derived from a multivariate model including age, sex, race, BMI, diabetes, hypertension, statin use, site, and smoking. HR (hazard ratio).

## Abbreviations

NAFLD	Nonalcoholic fatty liver disease
T2DM	Type 2 Diabetes mellitus
NASH	Nonalcoholic steatohepatitis
HCC	Hepatocellular carcinoma
MetS	Metabolic syndrome
NAFL	Nonalcoholic fatty liver
SNP’s	Single nucleotide polymorphisms
CVD	Cardiovascular disease
NAS	NAFLD activity score
TGF-β1	Transforming growth factor-beta1
